# The Impact of Hypoglycemic Therapy on the Prognosis for Acute Coronary Syndrome in Patients with Type 2 Diabetes

**DOI:** 10.3390/jpm12050845

**Published:** 2022-05-22

**Authors:** K. Yu. Nikolaev, A. I. Shevela, S. V. Mustafina, O. D. Rymar, A. K. Ovsyannikova, E. M. Zelenskaya, A. Y. Kovaleva, G. I. Lifshits

**Affiliations:** 1 Institute of Internal and Preventive Medicine—Branch of Institute of Cytology and Genetics, Siberian Branch of Russian Academy of Sciences, 175/1 Borisa Bogatkova Str., Novosibirsk 630089, Russia; nikolaevky@yandex.ru (K.Y.N.); svetlana3548@gmail.com (S.V.M.); orymar23@gmail.com (O.D.R.); aknikolae-va@bk.ru (A.K.O.); 2 Institute of Chemical Biology and Fundamental Medicine, Siberian Branch of the Russian Academy of Sciences, 8 Ak. Lavrentiev, Novosibirsk 630090, Russia; ashevela@mail.ru (A.I.S.); helenzlnsk@gmail.com (E.M.Z.); gl62@mail.ru (G.I.L.)

**Keywords:** acute coronary syndrome, type 2 diabetes, hypoglycemic therapy, prognosis

## Abstract

The article discusses particular circumstances of acute coronary syndrome (ACS) in patients with type 2 diabetes (T2D). In addition, the available literature data and clinical guidelines reflecting the role of hypoglycemic therapy as a cardioprotection factor in ACS are analyzed. The article considers possible protective molecular mechanisms of various groups of drugs in ischemic cardiomyocytes.

## 1. Introduction

Nowadays, cardiovascular diseases affect approximately 32.2% of all patients with type 2 diabetes (T2D) and are currently the main cause of death among people with type 2 diabetes [1]. Hyperglycemia and diabetes mellitus in patients with ACS are a strong predictor of poor prognosis for such patients [2]. It is a well-known fact that T2DM contributes significantly to cardiovascular morbidity and mortality. A large meta-analysis Emerging Risk Factors Collaboration (700,000 people) revealed that T2DM increases the risk of coronary heart disease, major stroke and death associated with other cardiovascular causes by two times [3]. Hyperglycemia on hospital admission is an important risk factor for hospital complications in patients with ACS. Blood glucose control with acute insulin infusion is especially important during the acute of post-ACS [4]. The impact of glycemic control on cardiovascular outcomes in patients with ACS and T2D remains an open question [5]. Moreover, the effects of type 2 diabetes mellitus medications on secondary prevention after ACS also remain unclear [6]. In this regard, the purpose of this review is to assess the impact of hypoglycemic therapy on the prognosis for ACS in patients with type 2 diabetes.

Risk factors for the development and progression of ACS are known, but the exact pathophysiological mechanisms underlying myocardial infarction (MI) in diabetes are unknown [7].

It is known that diabetes is a predictor of abnormal progression of ACS. The peculiarity of its clinical symptoms in patients with type 2 diabetes is a pain syndrome that is less severe than usual, which leads to abnormal symptoms or painless myocardial ischemia. This phenomenon can be explained by autonomic neuropathy, which leads to the increased threshold of ischemic pain perception [8]. Diabetic cardiomyopathy (DCM) is a definite complication of diabetes mellitus. The accumulated statistics showed that diabetes mellitus causes functional and structural changes in the heart, regardless of arterial hypertension, coronary artery atherosclerosis, or any other known heart disease, giving self-evident support for the existence of DCM [9]. One of the main theories of DCM causality is diabetic neuropathy, which leads to sympathetic ablation affecting myocardial blood flow (MBF), which affects its perfusion and results in the absence of symptoms such as chest pain [10].

Patients with abnormal clinical picture are less likely to have a diagnosis of confirmed MI at the time of admission to the hospital; and they have a higher level of in-hospital mortality than patients complaining about their chest pain. Despite abnormal symptoms, these patients do not differ substantially from those with ACS-type chest pain, increased levels of troponins or severity of coronary stenosis [8]. Mortality after ACS among patients with T2D is higher than that in patients without diabetes. Such difference may be related to the degree of atherosclerosis, as the progression of diabetes is characterized by endothelial dysfunction and changed energy metabolism, which cause atherosclerosis in medium- and large-bore arterial vessels, thus causing injuries to coronary and peripheral arteries. In addition, atherosclerotic plaques tend to appear at an earlier time interval and progress faster [11]. Also, a higher mortality rate among patients with T2D may be related to the size of the left ventricular remodeled area, as well as the incidence of severe ventricular arrhythmias [8]. It is known that insulin resistance in type 2 diabetes can lead to decreased regenerative potential of cardiac muscle cells after ischemia. However, it is not clear whether insulin resistance is a predictor of adverse outcomes in patients with T2D and ACS [12]. 

The metabolism is almost entirely based on fatty acids in a diabetic. In ACS with insulin resistance, the cellular metabolism of the heart becomes completely dependent on fatty acids, which increases the oxygen demand of the cells, followed by cell necrosis and apoptosis. This partly explains the higher mortality after myocardial infarction [13]. Apoptotic myocyte loss promotes progressive cardiac remodeling through left ventricular enlargement and interstitial fibrosis, which leads to an increase in type III collagen synthesis by cardiac fibroblasts [7]. 

Endothelial dysfunction in diabetic patients leads to increased proliferation of smooth muscle cells after vascular injury. Thus, patients with T2DM are most likely to experience restenosis after percutaneous coronary intervention. The process of restenosis begins very early (one to three months after coronary angioplasty) [14]. High admission glucose is associated with a more hemodynamically unstable patient group with myocardial infarction and larger infarct size and high early mortality. Elevated HbA1c in patient with ACS is associated with more adverse baseline characteristics and a more gradual higher mortality over time [15]. There is ongoing discussion that higher HbA1c is a potential indicator of in-hospital mortality in ACS patients. Also, a high level of HbA1 is a predictor of short-term mortality in patients with ACS without established DM and without DM [2]. The mechanism of association between HbA1c and increased mortality in patients with ACS is still unknown. Higher levels of HbA1c reflect excessive intracellular protein glycosylation in cardiac myocytes such as CaMKII [16]. Zhang et al. showed that the initial level of fibrinogen in plasma is associated with the level of HbA1c in patients with ACS [17]. As the reader might be aware, fibrinogen is an important part of coronary thrombosis. It confirms that hyperglycemia is associated with increased mortality rates during ACS. Thus, it remains unclear whether hyperglycemia is a marker or mediator of high mortality and whether treatment of hyperglycemia affects the well-being outcome [18].

Glucose control is an important factor in the management of ACS in diabetic patients. Patients with diabetes presenting with hyperglycemia (≥10–11 mmol/L [180–198 mg/dL]) in the setting of an acute MI have been found to have an increased risk of in-hospital mortality (odds ratio [OR], 1.7; 95% CI, 1.2–2.4) [19]. Kosiborod M et al., in a study of 16,871 patients with acute myocardial infarction, showed that patients with hospitalized glucose levels > 200 mg/dL had a higher risk of in-hospital mortality than patients with an average level (<110 mg/dL) (OR, 4.1; 95% CI, 1.81–9.26) [20]. Therefore, it is important to consider tight insulin glycemic control to be considered in ACS patients with significant hyperglycemia (>180 mg/dL) [21]. A tight glycemic control during an acute ischemic damage is associated with an increased regenerative potential of the myocardium [22]. Good glycemic control during cardiac rehabilitation after ACS has a positive effect on the increased oxygen consumption peak (VO2 peak) regardless of the antidiabetic therapy used [23]. Patients with type 2 diabetes after ACS are at an increased risk of recurrent cardiovascular events. The choice of optimal hypoglycemic therapy depends on the patient’s metabolic profile (severe insulin resistance and insulin deficiency). It is important to note that increased mortality due to cardiovascular events associated with hypoglycemia suggests the need to restrict long-term use of drugs that affect insulin secretion (sulfonylurea medications and insulin) [24].

One of the glucometabolic strategies in ACS is associated with the infusion of insulin, glucose and potassium (GIK). It was believed that the simultaneous administration of potassium and insulin reduces the risk of ventricular arrhythmias, and insulin facilitates the transport of potassium into the cell. Glucose provides a more efficient energy metabolism than free fatty acids or ketone bodies. However, recent large studies, CREATE-ECLA or OASIS-6 GIK, have shown that GIK infusion has no effect on mortality, cardiac arrest, or cardiogenic shock in ACS. Moreover, it has been shown that such therapy may even be harmful in the early stages of ACS due to hyperkalemia. Another important problem with the GIK concept is explained in the CREATE-ECLA study: despite insulin administration, mean glucose levels increased during therapy from 162 mg/dL (9 mmol/L) to 187 mg/dL (10.4 mmol/L) after 6 h of the introduction of GIK. Since glucose levels are independently associated with mortality, a glucometabolic strategy that raises her glucose levels and therefore goes in the wrong direction can be interpreted as a positive result for a testable hypothesis [25].

## 2. Discussion

The impact of glycemic control on cardiovascular outcomes and the best approach to treat hyperglycemia during and following an acute cardiovascular event remain a matter of debate in resent time [5].

In the treatment of ACS affected by type 2 diabetes, due consideration should be given to the fact that patients take a large number of various drugs. In the American cross-examination of 875 patients with type 2 diabetes, half of the respondents said they used to take at least seven prescription drugs, and 49% said they used to take two or more antihyperglycemic drugs. In addition to antihyperglycemic drugs, most often the respondents used to take antihypertensive drugs (71% of respondents) and drugs for hyperlipidemia (53%) [8]. Given these data, it is important to consider glycemic effects of cardioprotective drugs, as well as effects of antihyperglycemic drugs on the cardiovascular system. For instance, thiazolidinediones can cause fluid retention, thereby contributing to the development of congestive cardiac failure (CCF). The main clinical effects of diabetic drugs on the cardiovascular system and renal failure are shown in Table 1.

## 3. Metformin

Metformin is the most commonly prescribed antihyperglycemic drug of the biguanide class that is considered the “gold standard” for the treatment of T2D. At the moment, it is preferable to prescribe metformin as initial treatment for those patients who have not previously taken hypoglycemic therapy; and then they shall take metformin as long as possible. The American Association of Clinical Endocrinologists (AACE) and the American Diabetes Association (ADA) call metformin a drug of choice for glycemic control of diabetes mellitus [26]. Metformin reduces hyperglycemia, suppresses hepatic gluconeogenesis, and increases insulin sensitivity. Although the exact mechanism of metformin’s action is not completely understood, its ability to stimulate phosphorylation and, therefore, to activate the AMP-activated protein kinase (AMPK) is considered central to its mode of action and leads to inhibition of gluconeogenic genes [27].

Experimental data indicate metformin’s favorable effects on the left ventricular function. These effects are largely mediated by activation of the ATP-activated protein kinase, which plays a key role in such biochemical processes as glycolysis, oxidative phosphorylation of fatty acids, mitochondrial biogenesis, and glucose uptake. These processes contribute significantly to increase ATP levels and to restore myocardial contractility. AMPK also activates endothelial nitric oxide synthase (eNOS) and promotes autophagy, thereby preventing inflammation and necrocytosis. In diabetic patients with ST-elevation myocardial infarction (STEMI), retrospective analyses showed that their treatment with metformin is associated with reduced necrotic size as compared with patients not receiving metformin, implicating beneficial effects beyond glucose control. A recent GIPS-III randomized trial showed that metformin may improve left ventricular function following STEMI even in patients without diabetes [28]. However, nowadays there is no evidence of any randomized placebo-controlled trials to evaluate the efficacy in patients with type 2 diabetes and ACS. Single studies showed that metformin reduced all-cause mortality after 2-year follow-up and lowered the risk of recurrent CV events after five-year follow-up among patients with type 2 diabetes and ACS [6,29]. The survival benefit of metformin in patients with type 2 diabetes and ACS has been investigated in several retrospective analyses. Glyburide monotherapy increased the risk of death by 2.5-fold in patients with myocardial infarction undergoing emergency PCI compared with metformin. The study Jørgensen et al. had a small sample size in both groups (129 participants and 47 participants received metformin and glyburide therapy, respectively), and there was no covariant adjustment for renal function, T2D duration, HbA1c, serum LDL-C level, antiplatelet therapy, and smoking status [30]. Komaru et al. demonstrated what patients with ACS and T2D receiving metformin during the acute phase of their ACS event showed a significantly lower incidence of recurrent CVD compared with those who did not receive metformin [6]. Currently, metformin is contra-indicated in patients with diabetes mellitus and ACS due to the risk of tissue hypoxia and understudied effects on early and long-term clinical outcomes of ACS [57]. In addition, in case of ACS affected by type 2 diabetes, a rare but life-threatening adverse reaction (metformin-associated lactic acidosis) may occur [58]. However, some authors postulate that the cardioprotective effects of this medication may outweigh the risks in patients with ACS [6].

## 4. Sulfonylureas

The hypoglycemic effect of sulfonylurea drugs is based on the stimulation of pancreatic β-cells, which leads to increased endogenous insulin secretion. On the β-cell membrane, the drug molecule binds to the receptor associated with ATP-dependent potassium channels, which results in the β-cell insulin release. However, with prolonged treatment, stimulating effects of these drugs decline. It is assumed that this phenomenon is due to a drop in the number of receptors on the surface of β-cells [43]. In the available literature, we did not find any information on the effect of these drugs on the progress of ACS in type 2 diabetes. The effect of these drugs on the occurrence of cardiovascular events is not clearly known yet. The observational studies provide evidence for increased CV risks during treatment with sulfonylureas, while the results of randomized controlled trials indicate that there is no increase in risk of cardiovascular complications during treatment with sulfonylureas [24]. One possible explanation for such conflicting information is the fact that these drugs are bound together regardless of the fact that their pharmacological and pharmacokinetic properties have some differences. For example, gliclazide seems to bind selectively to pancreatic receptors, while glyburide binds non-selectively to pancreatic and myocardial receptors. Such bonds to myocardial receptors may reduce protective effects of ischemic conditioning. Therefore, the association of glyburide with cardiomyocyte sulfonylurea receptors may contribute to the extension of myocardial infarction, as well as reduction of the left ventricle functional activity [44].

## 5. DPP-4 Inhibitors

Dipeptidyl peptidase 4 inhibitors (DPP4i) are a class of drugs that prevent degradation of incretin hormones (i.e., glucagon-like peptide 1 (GLP1)) and increase insulin secretion, and also inhibit glucagon release [40]. The group of DPP4i drugs is heterogeneous by the nature of its effect on the cardiovascular system. For instance, sitagliptin reduces cardiac apoptosis and cardiac muscle fibrosis in renal hypertension. In addition, it contributes to autophagy, which has a protective effect on vascular endothelial cells. As a result of monitoring patients with type 2 diabetes after ACS, the frequency of repeated heart attacks, pulmonary edema, and acute renal failure at the hospital stage of treatment significantly decreased during treatment with sitagliptin. [41]. The ESPECIAL-ACS study showed that six-month treatment with sitagliptin helps to stabilize coronary atherosclerotic plaques in patients with type 2 diabetes after ACS [42].

## 6. Glucagon-Like Peptide 1 (GLP1) Agonists

An improvement in beta-cell function may be of prognostic importance in patients with MI. GLP-1 has beneficial effects on the myocardium by increasing myocardial glucose uptake, improving endothelial function and potentially reducing infarct size; it also has anti-inflammatory and anti-atherogenic properties [36]. The mechanism of GLP-1 is associated with stimulation of insulin secretion and suppression of glucagon in a glucose-dependent manner. GLP1 receptors agonists are a class of parenteral hypoglycemic drugs that activate GLP1 endogenous incretin receptors. The mechanism of effect of this group is based on inhibition of glucagon secretion, ensuring insulin release in response to hyperglycemia, also leading to delayed gastric emptying, which provides for enhanced satiety. These drugs have an anti-inflammatory effect in blood vessels, have an antihypertensive action, improve endothelial function, and have an experimentally determined effect on reducing myocardial infarction size [37]. Glycemic variability (GV) is an independent cardiovascular risk factor in patients with ACS. Glucose levels below 90 mg/dL (5 mmol/L) and above 180 mg/dL (10 mmol/L) should be avoided in patients with an acute coronary syndrome. In addition, it is important not only to maintain glucose levels within these values, but also to reduce short-term GV, since it is associated with an increase in MACE in these patients. Describe the beneficial effect of GLP-1 agonists in short-term GV and oxidative stress in the initial period of ACS [38]. The ELIXIA trial evaluated the effect of GLP1 agonist lixisenatide on cardiovascular outcomes in patients with type 2 diabetes after ACS. The trial determined that the use of this drug had little impact on the prognosis in patients with T2D and ACS, compared to the placebo control [39].

## 7. Insulin Therapy

Randomized studies demonstrated inconsistent results regarding the benefit of intravenous insulin for glycemic control in patients with ACS [45].

In the heart, insulin controls glucose transport, glycolytic speed, glycogen synthesis, growth, contractility of cardiomyocytes and their survival, acting mainly through insulin receptor subunit (IRS-1/2) proteins, phosphoinositide-3-kinase (PI3K), Akt (5) and the mammalian target of rapamycin (mTOR) signaling pathway [46]. Hyperglycemia in ACS leads to increased oxygen consumption by the heart cells and a more severe ischemic condition. Insulin lowers glucose levels and limits the bad effects of hyperglycemia. In addition, insulin utilizes myocardial glucose and reduces the concentration of free fatty acids due to the inhibitory effect of insulin on lipolysis [47]. The introduction of insulin protects the myocardium in the ischemic and reperfusion phases. Insulin administration creates conditions for an “optimal environment” by the effects of glucose, the anti-inflammatory action of insulin, and its action on NO synthesis [13]. The hypoglycemia in the insulin-treated patients may be having an adverse effect. Several studies have associated hypoglycemia (plasma glucose ≤ 3.9 mmol/L) with an increase in cardiovascular mortality, including those following an ACS [48]. Hypoglycemia often occurs during insulin therapy (10–22%). Hypoglycemia has adverse physiological effects: hypercoagulability, inflammatory response, and QT interval prolongation [49]. In patients with T2D, in response to hypoglycemia, the sympathoadrenal system is triggered, which has hemodynamic, hemostatic, hemorheological, and electrophysiological effects, which can lead to myocardial ischemia, arrhythmias and sudden death. Therefore, in the treatment of such patients with ACS, insulin is recommended to use only when strictly necessary [50]. However, some investigators demonstrate that basal-bolus correction insulin regimen with glycemic target < 200 mg/dL in patients with ACS could lead to a hypoglycemic risk very close to zero (0.24%), with a significant reduction in hypoglycemia-related clinical events [51]. Some scientific societies agree about the need for intravenous insulin therapy with concomitant infusion of glucose solution to target a blood glucose level of 1.40–1.80 g/L [52,53]. The aim of the DIGAMI study was to investigate the long-term prognosis in diabetic patients with ACS receiving continuous intravenous (IV) insulin for glucose control [54]. Insulin-glucose infusion was carried out within 24 h after hospitalization and subsequent insulin therapy after discharge. The annual mortality was 18.6% in the infusion group compared to 26.1% in the control group. The main benefit was seen during the early post-infarction period, namely a trend of reduced rates of heart failure [55]. Subsequent reports confirmed these observations, showing an absolute 11% reduction in long-term mortality (three to four years) in patients allocated to insulin-glucose infusion. The best outcomes were in low-risk patients with high admission blood glucose and with no prior insulin treatment [25]. Later, the DIGAMI 2 study (1253 patients) compared the three treatment protocols: (1)-infusion of insulin and glucose within 24 h after hospitalization, followed by long-term insulin therapy; (2) infusion of insulin-glucose followed by standard glucose control; and (3) standard metabolic treatment according to local practice. The DIGAMI 2 study did not support the hypothesis that an insulin treatment strategy reduces mortality in type 2 diabetic patients after MI. There is an explanation for this result. Thus, the overall mortality in DIGAMI 2 was lower than expected. This may be since concomitant treatments were performed better than in the first study. Thus, 70% of patients were treated with statins in the second trial and only 8% in DIGAMI 1. In addition, the three strategies did not result in a significant difference in glucose control among patients. Target glucose levels were not achieved in group 1. However, the levels in all groups were significantly better than in DIGAMI 1. The study had to be terminated early due to a slow recruitment rate, resulting in a drop in statistical power below 50% [59]. Currently, the effectiveness of combination therapy of insulin with other antihyperglycemic drugs in patients with ACS for the prevention of hypoglycemia is being studied. There is evidence that adding vildagliptin (DPP4i) to insulin therapy during ACS can significantly reduce hypoglycemic events while maintaining acceptable blood glucose levels [60].

## 8. Sodium Glucose Transporter Inhibitors (SGLT-2)

In the available literature, we did not find any information on the effect of these drugs having glycosuric effect on the prognosis of ACS in in diabetics [32]. However, the EMPA-REG study demonstrated that treatment with the SGLT-2 inhibitor empagliflozin in combination with metformin reduces cardiovascular mortality by 38% in patients with T2D and high cardiovascular risk [33]. Dapagliflozin has been shown to be effective in reducing the risk of hospitalization for chronic heart failure (CHF) in patients with type 2 diabetes in the study DECLARE TIMI 58. DAPA-HF is the first clinical study in which dapagliflozin was prescribed not for the purpose of correcting diabetes but for the treatment of CHF with low ejection fraction [34]. Dapagliflozin reduces glucose reabsorption goats, increases its excretion in the urine, which leads to a decrease in the concentration of glucose in the blood cheek and after meals. Dapagliflozin has no effect on endogenous glucose production. The beneficial effect of SGLT-2 on the cardiovascular system and kidneys can be explained by glucosuria and natriuresis (decrease in plasma volume, pre-and afterload on the heart, decrease in blood pressure and arterial wall stiffness, decrease in intraglomerular pressure and glomerular hyperfiltration) [35].

## 9. Conclusions

Thus, for the present, the problem of using hypoglycemic therapy to reduce the risk of cardiovascular complications in patients with T2D and ACS persists. The effect of metformin on early and long-term clinical outcomes of ACS is not well known, insulin therapy is associated with potentially dangerous hypoglycemia in ACS, and the use of other antihyperglycemic drugs to prevent cardiovascular events in the above category of patients remains understudied.

## Figures and Tables

**Table 1:** Clinical factors in choosing the main hypoglycemic drugs.

Basic Hypoglycemic Drugs	Cardiovascular Effects		Renal Effects		References
	ACD	Heart Failure	Chronic Kidney Disease	Contraindications	
Metformin	Potential benefits	Neutral	Neutral	Contraindicated at GRF < 30 mL/min/1.73 m^2^	[6,26,27,28,29,30,31]
SGLT-2 inhibitors	Benefit	Benefit: dapagliflozin empagliflozin	Benefit: dapagliflozin	Contraindicated at GRF < 30 mL/min/1.73 m^2^	[32,33,34,35]
GLP1 agonists	Benefit: liraglutide	Neutral	Benefit: liraglutide	Contraindicated at GRF < 30 mL/min/1.73 m^2^	[36,37,38,39]
DPP-4 inhibitors	Neutral	Potential risk: saxagliptin, alogliptin	Neutral	Dose adjustment required in renal failure	[40,41,42]
Sulfonylureas	Neutral	Neutral	Neutral	Glibenclamide is not recommended	[24,43,44]
Insulin	Neutral	Neutral	Neutral	Dose adjustment required in renal failure	[13,25,45,46,47,48,49,50,51,52,53,54,55]
Thiazolidinediones	Potential benefits: pioglitazone	Increased risk	Neutral	Not recommended in renal failure due to risk of fluid retention.	[56]

ACD, associated cardiovascular diseases; GFR, glomerular filtration rate.
